# Identification of the high-risk group for metastasis of gastric cancer cases by *vascular endothelial growth factor receptor-1* overexpression in peripheral blood

**DOI:** 10.1038/sj.bjc.6603785

**Published:** 2007-05-08

**Authors:** Y Kosaka, K Mimori, T Fukagawa, K Ishikawa, T Etoh, H Katai, T Sano, M Watanabe, M Sasako, M Mori

**Affiliations:** 1 Department of Surgery, Medical Institute of Bioregulation, Kyushu University, 4546, Tsurumihara, Beppu 874-0838, Japan; 2 Gastric Surgery Division, National Cancer Center Hospital, 5-1-1 Tsukiji, Chuo-ku 104-0045, Japan; 3 Department of Surgery, Kitasato University School of Medicine, 1-15-1 Kitasato, Sagamihara 228-8555, Japan

**Keywords:** gastric cancer, peripheral blood, bone marrow, vascular endothelial growth factor receptor-1, metastases

## Abstract

Identification of an isolated tumour cell with metastatic ability is important for predicting the recurrence and prognosis of gastric cancer. A biological marker for evaluating the metastatic ability of gastric cancer cells has not yet been identified. We assessed *vascular endothelial growth factor receptor-1* mRNA expression by quantitative real-time reverse transcriptase-polymerase chain reaction. *Vascular endothelial growth factor receptor-1* mRNA in peripheral blood was more highly expressed in perioperative metastasis-positive and postoperative recurrence cases than in normal control cases, early cancer cases and nonmetastatic advanced cancer cases. The peripheral blood *vascular endothelial growth factor receptor-1* mRNA-positive group was associated with advanced clinical stage, deep invasion beyond the muscularis propria, lymphatic involvement, vascular involvement, lymph node metastasis, positive peritoneal lavage cytology, preoperative metastasis and postoperative recurrence. Flow cytometry analysis disclosed that vascular endothelial growth factor receptor-1 expressing cells in the peripheral blood were more abundant in cancer cases with metastases than in cases without metastases. Our data suggest that the amount of positive cells may provide information on the clinical features of gastric cancer, especially in regard to gastric cancer metastasis.

Vascular endothelial growth factor (VEGF) plays an important role in cancer progression. The receptors of VEGF consist of VEGFR1 (Flt-1), VEGFR2 (KDR/Flk-1) and VEGFR3 (Flt-4). VEGFR1 is a receptor not only for VEGF, but also for placental growth factor (PGF), and is associated with tumour progression and neovascularization (Pajusola *et al*, 1992; Galland *et al*, 1993; Ferrara & Davis-Smyth, 1997; Shibuya *et al*, 1999; Hiratsuka *et al*, 2001). Furthermore, there are splicing variants from the original VEGFR1 protein. Kendall and Thomas (1993) reported that soluble VEGFR1 (sVEGFR1) is one of the splicing variants derived from the membrane penetrating type VEGFR1 protein, and therefore there is considerable difference between the soluble protein and the one in this study. The sVEGFR1 competitively inhibits the binding between VEGFR1 and its ligands, such as VEGF and PGF. Moreover, Clark *et al* (1998) reported that sVEGFR1 exists specifically in serum from pregnant women, and does not exist in serum from men and nonpregnant women. For that reason we did not assess sVEGFR1 protein in this study.

Recently, Kaplan *et al* (2005) reported that bone marrow progenitor cells expressing VEGFR1 play an important role in the development of malignant metastatic foci. Their finding suggests that VEGFR1-expressing cells in the bone marrow or peripheral blood may be related to cancer metastasis and recurrence. There have been no reports studying the clinical and pathological significance of *VEGFR1* mRNA expression in the bone marrow and peripheral blood of cancer patients up to the present time. We have therefore studied its significance in gastric cancer patients, and have demonstrated that patients with high *VEGFR1* mRNA expression in bone marrow or peripheral blood have significantly higher metastasis and recurrence rates than those with low *VEGFR1* mRNA expression.

## MATERIALS AND METHODS

### Patients

Ninety gastric cancer patients who underwent surgical treatment in the National Cancer Center Hospital, Japan, from 2001 to 2004 were enrolled in the study. Sixteen patients with no history of cancer, who underwent abdominal operation in our hospital from 2001 to 2004, were recruited as noncancer controls. The mean postoperative period was 9.8 months, ranging from 4 to 24 months. The clinical stages and pathologic features of primary tumours were defined according to the criteria of the Japanese classification of gastric carcinoma (Japanese Research Society for Gastric Cancer, 1999). The ages of the 68 male and 38 female patients ranged from 31 to 85 years. Written informed consent was obtained from all patients. The total 106 patients were divided into the following four groups: group 1 consisted of noncancer patients (*n*=16); group 2 consisted of early cancer patients, with tumours that had invaded less than the entire submucosal layer (*n*=30); group 3 consisted of advanced cancer patients, where there was evidence of deep invasion beyond the muscularis propria and no preoperative distant metastasis (*n*=30); and group 4 comprised patients with metastasis and recurrence, where there were distant metastases at the time of operation (i.e., liver and/or lung metastasis, peritoneal dissemination) (*n*=18), or, patients up to April 2005, who developed postoperative recurrence (i.e., peritoneal dissemination and distant metastasis) (*n*=12). Additionally, the 18 gastric cancer patients with metastases at the time of operation had palliative therapies (gastrointestinal reconstruction and control of bleeding), to improve patient quality of life.

### Bone marrow and blood sampling

Aspiration of both peripheral blood and bone marrow was conducted under general anaesthesia immediately before surgery. The bone marrow aspirate was obtained from the sternum using a bone marrow aspiration needle (MDTECH, Gainesville, FL, USA), and peripheral blood was obtained through a venous catheter. The first 1 ml of both peripheral blood and bone marrow was discarded to avoid contamination by epidermal cells. A 1 ml sample of peripheral blood and bone marrow was each immediately mixed vigorously with 4 ml of ISOGEN-LS (NIPPON GENE, Toyama, Japan) and stored at −80°C until RNA extraction.

### Total RNA extraction and first-strand cDNA synthesis

Total RNA was extracted according to the ISOGEN-LS manufacturer's protocols. All the clinical samples obtained from the National Cancer Center Hospital were sent to our institute. The reverse transcriptase reaction (RT) was performed as described previously (Mori *et al*, 1995; Masuda *et al*, 2002). First-strand cDNA was synthesized from 2.7 *μ*g of total RNA in a 30 *μ*l reaction mixture containing 5 *μ*l 5 × RT reaction buffer (BRL, Gaithersburg, MD, USA), 200 *μ*M dNTP, 100 *μ*M solution random hexadeoxynucleotide primer mixture, 50 U of RNasin (Promega, Madison, WI, USA), 2 *μ*l 0.1 M dithiothreitol and 100 U of Molony murine leukaemia virus RT (BRL). The mixture was incubated at 37°C for 60 min, heated to 95°C for 10 min, and then chilled on ice.

### Quantitative RT–PCR

The sequences of *VEGFR1* mRNA were as follows: sense primer, 5′-TCATGAATGTTTCCCTGCAA-3′; and antisense primer, 5′-GGAGGTATGGTGCTTCCTGA-3′. Ribosomal protein S27a (RPS27A) was used as an internal control. The sequences of RPS27A primers were as follows: sense primer, 5′-TCGTGGTGGTGCTAAGAAAA-3′; and antisense primer, 5′-TCTCGACGAAGGCGACTAAT-3′. Real-time monitoring of PCR reactions was performed using the LightCycler™ system (Roche Applied Science, Indianapolis, IN, USA) and SYBR green I dye (Roche Diagnostics). Monitoring was performed according to the manufacturer's instructions, as described previously (Masuda *et al*, 2003; Ogawa *et al*, 2004). In brief, a master mixture was prepared on ice, containing 500 ng of cDNA of each gene, 2 *μ*l of LC DNA Master SYBR green I mix, 50 ng of primers and 2.4 *μ*l of 25 mM MgCl_2_. The final volume was then adjusted to 20 *μ*l with water. After the reaction mixture was loaded into the glass capillary tube, PCR was carried out under the following cycling conditions: initial denaturation at 95°C for 10 min, followed by 40 cycles of denaturation at 95°C for 8 s, annealing at 68°C for 8 s and extension at 72°C for 2 s. After amplification, the products were subjected to a temperature gradient from 72 to 95°C at 0.1°C/s, under continuous fluorescence monitoring to produce a melting curve of the products.

### Flow cytometry analysis

To determine VEGFR1 protein expression in peripheral blood, we obtained preoperatively 10 ml of heparanized peripheral blood from gastric cancer patients with or without distant metastasis. Blood mononuclear cells were obtained by Ficoll density centrifugation at 1500 **g** for 30 min. Phycoerythrin-conjugated monoclonal antibody against human VEGFR1 was purchased from R&D Systems (Minneapolis, MN, USA). Mononuclear cells (1 × 10^6^) were stained with VEGFR1 antibody after Fc receptor blocking, and analysed by the BD FACS Vantage™ SE flow cytometry system. The data were analysed using CellQuest soft ware (BD Biosciences, San Jose, CA, USA).

### Data analysis

The expression levels of *VEGFR1* and *RPS27A* mRNA were determined by comparison with the cDNA from Human Universal Reference Total RNA (Clontech, Palo Alto, CA, USA). After proportional baseline adjustment, the fit point method was employed to determine the cycle in which the log-linear signal was first distinguishable from the baseline, and then that cycle number was used as a crossing-point value. The standard curve was produced by measuring the crossing point of each standard value and plotting it against the logarithmic value of the concentrations. Concentrations were calculated by plotting their crossing points against the standard curve, and were adjusted by *RPS27A* content. Taking into consideration the clinical application of the current study, the 95% confidence interval was used as the upper limit of a normal case cutoff value (bone marrow, 0.12; peripheral blood, 0.059). The 95% value of a normal case according to the reference intervals of the Clinical and Laboratory Standards Institute (Sasse, 2000) was established, and the reference limit was regarded as the cutoff value. Levels higher or lower than the cutoff value were considered ‘positive’ and ‘negative’, respectively. The sensitivity and specificity of the data were determined to evaluate the legitimacy of the cutoff value.

### Statistics

For continuous variables, the data were expressed as the mean±s.d. The relationships between *VEGFR1* mRNA expression and the clinicopathological factors were analysed using the *χ*^2^-test and Kruskal–Wallis test. All tests were analysed using JMP software (SAS Institute Inc., Cary, NC, USA). Statistical significance was determined from two-sided tests as *P*<0.05.

## RESULTS

### Expression of *VEGFR1* mRNA in peripheral blood and bone marrow of surgical gastric cancer patients

**Figure 1:**
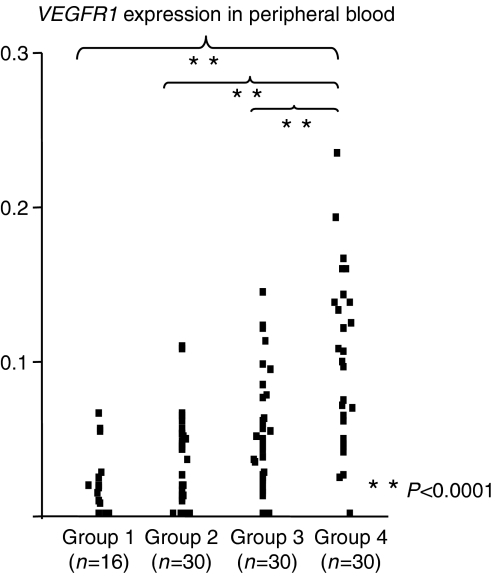
*VEGFR1* mRNA expression in peripheral blood from gastric cancer cases. Group 1 consisted of noncancer patients (*n*=16). Group 2 consisted of early cancer patients, with tumours that invaded less than the sub-mucosal layer (*n*=30). Group 3 consisted of advanced cancer patients, where there was evidence of deep invasion beyond the muscularis propria and no preoperative distant metastasis (*n*=30). Group 4 was the metastasis and recurrence patient group, where there were distant metastases at the time of operation (i.e., liver and/or lung metastasis, peritoneal dissemination) and who developed postoperative recurrence (e.g., peritoneal dissemination and distant metastasis) (*n*=30). The mean expression level of *VEGFR1* mRNA in group 4 was significantly higher than all other groups (*P*<0.0001, respectively).

**Figure 2:**
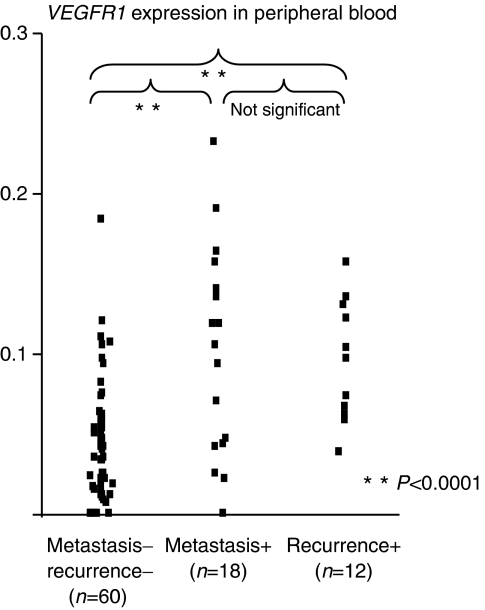
*VEGFR1* expression with or without metastasis and recurrence. There is no significant difference between patients with metastasis at the time of operation and patients with postoperative recurrence. The higher *VEGFR1* expression was observed in patients with metastasis pre- and postoperatively in comparison with gastric cancer patients without metastasis (*P*<0.0001, respectively).

Figure 1 shows peripheral blood *VEGFR1* mRNA levels of the four groups. In peripheral blood, the mean expression level of *VEGFR1* mRNA in group 4 (0.099±0.055) was significantly higher than all other groups (*P*<0.0001; group 1 (0.033±0.05), group 2 (0.044±0.039) and group 3 (0.045±0.039)). It is of note that there was no significant difference in *VEGFR1* expression levels in the 18 cases with metastasis at the time of operation compared with the 12 cases with postoperative recurrence; however, 30 cases with recurrence/metastasis expressed a significantly higher level of *VEGFR1* than the 60 gastric cancer cases without metastasis/recurrence (Figure 2). In bone marrow samples, there was no clear relationship between the expression level of *VEGFR1* mRNA and the progression of gastric cancer cases.

### *VEGFR1* expression and clinicopathological features of gastric cancer patients with surgery

The correlations between the results for the *VEGFR1* mRNA levels and clinicopathological variables are summarized in Table 1. By the predetermined cutoff values for bone marrow, 0.12, and peripheral blood, 0.059, of the 90 patients there were 23 (25.6%) and 34 (37.8%) estimated to be positive for *VEGFR1* mRNA in bone marrow and peripheral blood, respectively. Sensitivities with these cutoff values were 66.7% in peripheral blood and 46.7% in bone marrow, and specificities were 76.7% in peripheral blood and 85.0% in bone marrow. In the peripheral blood, a significantly higher number of *VEGFR1* mRNA positive cases belonged to the following clinical subgroups: those in stages 3 and 4 (*P*<0.001), invasion deeper than the muscularis propria (*P*<0.01), lymphatic involvement (*P*<0.001), vascular involvement (*P*<0.0001), lymph node metastases (*P*<0.0001), positive peritoneal lavage cytology (*P*<0.001), perioperative overt metastases (e.g., liver or lung *P*<0.05) and postoperative recurrence (*P*<0.01). In contrast, in bone marrow, there was a significant difference observed that correlated with the pathological stage (*P*<0.05), the incidence of lymphatic involvement (*P*<0.05), lymph node metastases (*P*<0.01), positive peritoneal lavage cytology (*P*<0.05) and the presence of postoperative recurrence (*P*<0.01).

### VEGFR1 expression in blood by flow cytometry

**Figure 3:**
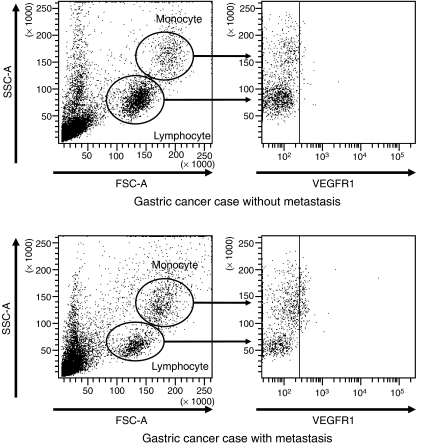
*VEGFR1* expression in blood of gastric cancer cases with or without metastasis. The number of *VEGFR1*-positive cells in a representative gastric cancer case with metastasis was more abundant (9.8%) than a case without metastasis (2.5%). Particularly, *VEGFR1*-positive cells in the fraction of monocytes in FS/SS plots were more abundant than those cells in the fraction of lymphoid cells.

According to flow cytometry analysis, VEGFR1-positive cells in the lymphocytes and monocytes of mononuclear cells isolated from the peripheral blood of gastric cancer patients with metastasis were increased over patients without metastasis (9.8. *vs* 2.5% in representative study case) (Figure 3). In particular, VEGFR1-positive cells in the fraction of monocytes in FS/SS plots were more abundant in the metastatic patient (9.7%) than in the nonmetastatic patient (2.4%).

## DISCUSSION

In this study, we studied *VEGFR1* mRNA expression in the bone marrow and peripheral blood of patients with gastric cancer. Patients with metastases and/or recurrence expressed higher levels of *VEGFR1* mRNA in the peripheral blood than nonmetastatic and nonrecurring patients. Patients with a high level of *VEGFR1* mRNA expression in peripheral blood showed deeper tumour invasion in the primary organ, positive vascular vessel or lymphatic vessel invasion, positive lymph node metastasis and positive peritoneal lavage cytology. Thus, the expression of *VEGFR1* mRNA in the peripheral blood may be associated with metastasis and recurrence of gastric cancer.

The source of *VEGFR1* mRNA in peripheral blood or bone marrow in gastric cancer patients has not yet been elucidated. We initially speculated that the original cells expressing VEGFR1 in gastric cancer patients are the haematopoietic progenitor cells (HPCs). Lyden *et al* (2001) reported that the progression of tumour vessels needs the cooperation of VEGFR1-positive HPCs and VEGFR2-positive endothelial progenitor cells. In addition, Kaplan *et al* (2005) reported that bone marrow-derived HPCs express VEGFR1 home to tumour-specific premetastatic sites, and form cellular clusters that provide a permissive niche for incoming tumour cells before the arrival of tumour cells. Another possible source may be mature vessel-derived endothelial cells, which might be the largest source of VEGFR1-expressing circulating endothelial cells (CECs) (Mancuso *et al*, 2001; Beerepoot *et al*, 2004). Contrary to our expectation, there was no significant difference in the number of VEGFR1-expressing cells of CD133^+/−^ CD31^+^ cells except the monocytes between a gastric cancer patient with metastasis and a patient without metastasis (data not shown). Our present study revealed that the original cells producing VEGFR1 may be monocytes. Bone marrow and peripheral blood samples contain many white blood cells, including monocytes, in addition to a few circulating CECs or HPCs. In patients with advanced cancer, IL-10 or IL-12 are expressed more frequently than in patients with early cancer or normal volunteers (Sugai *et al*, 2004). These findings indicate that the monocyte–macrophage lineage is activated in patients with advanced cancer, and *VEGFR1* may be expressed by this lineage (Sawano *et al*, 2001). In general, the metastatic pathway in gastric cancer is not haematogenous, and therefore, the responsible cells expressing VEGFR1 may be monocytes and not HPCs and CECs. A last possibility is that VEGFR1 originates from circulating cancer cells in the bone marrow or peripheral blood (Fan *et al*, 2005; Yang *et al*, 2006). However, the number of circulating cancer cells is very low, which allow us to ignore the possibility that cancer cell are the origin of VEGFR1 expression.

To our knowledge, this study is the first to describe the detection of *VEGFR1* mRNA in the circulating blood of cancer patients. It will be very important to determine in advanced cases which cells produce *VEGFR1* mRNA in the peripheral blood. In brief, using flow cytometry analysis to detect VEGFR1-expressing cells in blood, we found that the number of VEGFR1-positive cells in peripheral blood was distinctly larger in a gastric cancer case with metastasis than in a case without metastasis. In gastric cancer cases with metastases, VEGFR1-positive monocytes were more abundant than the other cells, including CECs and HPCs. Because there are much fewer CECs and HPCs than monocytes, the *VEGFR1* mRNA which we detected in this study may be of monocyte origin. That VEGFR1-positive HPCs and CECs may also contribute to gastric cancer progression has been supported by several reports. Additional study is needed to verify the function of individual VEGFR1-expressing cells.

In conclusion, the evaluation of *VEGFR1* mRNA in the peripheral blood samples of gastric cancer patients could be very important, because it may be a valuable marker for cancer metastasis or recurrence. When considering the clinical application of this marker, it is a fortuitous finding, because from a practical standpoint, it is easier to obtain peripheral blood samples than bone marrow samples. In addition, our final goal will be to evaluate the protein level of VEGFR1 in blood samples of cancer patients, to determine its practical use as a tumour marker.

## Figures and Tables

**Table 1:** Relationship between clinicopathological variables and the VEGFRI mRNA expression in peripheral blood bone and marrow from gastric cancer cases

	***VEGFR1* -bone marrow**				***VEGFR1* -peripheral blood**			
	**Positive**		**Negative**		**Positive**		**Negative**	
**Features**	***n***	**(*n*=23)**	**(*n*=67)**	***P* value**	***n***	**(*n*=34)**	**(*n*=56)**	***P*-value**
*Sex*								
Male	61	16	45	0.83	61	26	35	0.17
Female	29	7	22		29	8	21	
Age (mean±s.d.)		62.7±13.4	59.6±11.1	0.14		62.5±11.2	59.2±12	0.1

*Stage*								
1 and 2	55	9	46	<0.05	55	13	42	<0.001
3 and 4	35	14	21		35	21	14	

*Invasion depth*								
Slighter than submucus	28	4	24	0.09	28	5	23	<0.01
Deeper than muscularis propria	60	19	43		62	29	33	

*Lymphatic involvement*								
Negative	58	10	48	<0.05	58	14	44	<0.001
Positive	32	13	19		32	20	12	

*Vascular involvement*								
Negative	65	14	51	0.16	65	16	49	<0.0001
Positive	25	9	16		25	18	7	

*Lymph node metastasis*								
Negative	62	10	52	<0.01	62	15	47	<0.0001
Positive	28	13	15		28	19	9	

*Peritoneal lavage cytology*								
Negative	71	14	57	<0.05	71	20	51	<0.001
Positive	19	9	10		19	14	5	

*Peritoneal dissemination*								
Negative	82	20	62	0.42	82	30	52	0.46
Positive	8	3	5		8	4	4	

*Perioperative overt metastasis*								
Negative	72	16	56	0.15	72	23	49	<0.05
Positive	18	7	11		18	11	7	

*Postoperative recurrence*								
Negative	78	16	62	<0.01	78	25	53	<0.01
Positive	12	7	5		12	9	3	

s.d.=standard deviation.
